# Strategies for clone detection, selection and isolation in Per.C6 cells - case for Rebmab100

**DOI:** 10.1186/1753-6561-7-S6-P38

**Published:** 2013-12-04

**Authors:** Fernanda P Yeda, Mariana L dos Santos, Lilian R Tsuruta, Bruno B Horta, André L Inocencio, Oswaldo K Okamoto, Maria C Tuma, Ana M Moro

**Affiliations:** 1 Lab. Biofármacos em Células Animais, Instituto Butantan, SP, 05503-900, Brazil; 2 Recepta-biopharma, SP, 04533-014, Brazil; 3 Depto. Genética e Biologia Evolutiva, Instituto de Biociências, Universidade de São Paulo, SP, 05508-900, Brazil

## Background

A successful monoclonal antibody (mAb) cell line development requires efficient clone detection and screening. Cloning by limiting dilution (LDC) is the traditional method to isolate mAbs expressing clones [1]. Although effective, LDC is time-consuming, with limited workflow and therefore a critical step of cell line development. To compare to LDC in terms of timelines and productivities for Rebmab100 mAb cell line development we have implemented ClonePix FL (CP-FL), an automated system for high throughput clone detection. The robotic colony picker has the advantages of reducing the process time and increasing the probability to isolate high-producing clones. Moreover, we have combined these two approaches with high throughput screening assays for early detection of high productive clones.

Rebmab100 mAb targets Lewis-Y, a blood group-related antigen expressed in over 70% of epithelial cancers, including breast, colon, ovary and lung carcinomas. The murine monoclonal 3S193 was generated in BALB/c mice by immunization with Le^y^-expressing cells from the MCF-7 breast carcinoma cell line [2]. The humanized version of anti- Le^y ^3S193 mAb was obtained by CDR-grafting method [3]. The hu3S193 (Rebmab 100) mAb has potent immune effector function (ADCC and CDC), is rapidly internalized into Le^y ^expressing cancer cells, and has been shown to cause significant regressions in xenograft models in preclinical studies, alone or in conjunction with isotope and toxins [3,4]. Safety and desirable pharmacokinetic profiles of Rebmab100 were demonstrated in a Phase I clinical trial in patients with epithelial carcinomas [5] and promising results have been obtained in a Phase II clinical trial conducted in Brazil [6]. Very importantly, Rebmab100 was granted orphan-drug status by the FDA for ovary cancer. Aiming the next step of Rebmab100 mAb development we generated a new Rebmab100 cell line that shows stability and high productivity allowing its scale-up to later clinical trials.

## Materials and methods

Suspension Per.C6^® ^cells (Crucell, Netherlands) were transfected with a vector containing the genes coding for heavy and light chains of Rebmab100 mAb. After selection by G418 the cells from the stable pool were cloned by limiting dilution or plated in semi-solid medium (Molecular Devices, USA) for ClonePix FL screening.

Cellular growth was assessed in plates, 96, 24 or 6-well plates, either by CloneSelect Imager (Molecular Devices) or Guava EasyCyte cytometer (Merck-Millipore). Antibody titers were measured by Biacore T100 (GE Healthcare, Sweden). The selected clones were transferred to T-flasks and subsequently to shaker flasks (SF). Clones were analyzed in 50 mL and 200 mL SF fed-batch processes. The stability study was performed for at least 50 generations in continuous culture and also starting batch runs with cells taken at different generations.

## Results

### Generation of Rebmab100 stable pool

The transfection of Per.C6^® ^cells with a vector containing the genes coding for heavy and light chains of Rebmab100 generated a stable pool through G418 selection.

### Cloning using two different approaches

The stable pool was cloned by LDC in liquid medium at 0.5 cell/well in 50 96-well plates, resulting in 261 colonies transferred to 24-well plates in 3-4 weeks after screening with the CloneSelect Imager. Concomitantly the same pool was seeded at different concentrations (300 to 2000 cells/mL) in semi-solid medium. The plates were screened by light and fluorescence images about ten days after seeding. A total of 845 colonies were picked, from which 225 were transferred to 24-well plates. At the transference step to 24-well plates, 261 out of 4800 wells seeded in LDC were transferred while 225 colonies out of 845 colonies picked by CP-FL, representing 5.4% and 26.6% efficiency, respectively. Both approaches followed sequential steps as transfer of the clones to 6-well plates, T-flasks and SF, selecting them at each step for cell growth and productivity related to cell number.

### Fed-batch experiments and stability study

**Figure 1:**
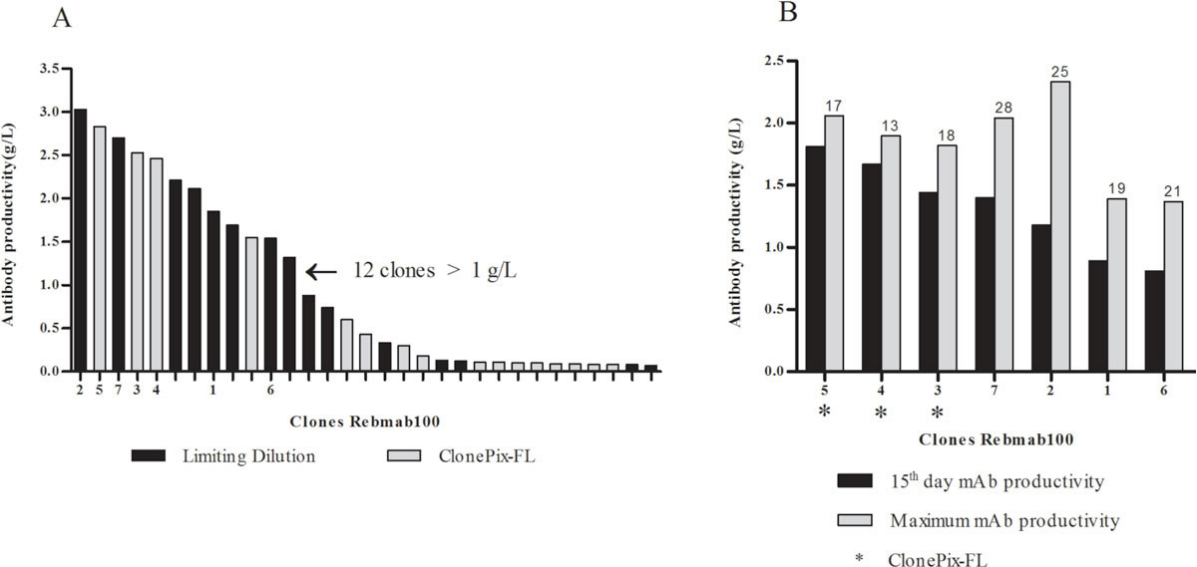
**Antibody titer measured by Biacore in SF fed-batch process (g/L)**. **(A) **31 selected Rebmab100 clones measured on the last day of a 50 mL SF fed-batch culture. **(B) **Maximum (grey bars) and 2 weeks (black bars) mAb productivity obtained in a 200 mL SF fed-batch culture for the 7 stable Rebmab100 clones. The number above the grey bars indicates the day when maximum mAb productivity occurred.

Thirty-one clones adapted to suspension cultures were assessed for productivity in fed-batch processes, being 15 originated from LDC and 16 from CP-FL. From the fed-batch in 50 mL SF 12 clones presented titers ranging from 1.3 to 3.0 g/L (Figure 1A). Out of 31 clones, 10 were selected for long-term stability study to determine growth and productivity along the time required for mAb production during a manufacturing process. The stability study performed with 6 LDC and 4 CP-FL originated clones ruled out 3 of them, two from LDC and one from CP-FL. Seven clones showed genetic and cellular stability (data not shown), 4 from LDC and 3 from CP-FL and were further analyzed in fed-batch in 200 mL SF. In this study we compared titers obtained after 2 weeks run for all clones, with results ranging from 0.9 to 1.8 g/L to the maximum productivity attained by each clone, obtained at different lengths of culture (Figure 1B). Taken together the data for cell growth, productivity, kinetic and functional assays of the purified antibodies (data not shown), mainly the immune-effector activity characteristically displayed by Rebmab100, we identified 4 lead clones, the first and second originated by CP-FL screening. Final ranking will be evaluated after bioreactor runs.

## Conclusions

The CP-FL automated picking has the advantage of being less labor-intensive and time-consuming, while allowing the chance of picking clones that would not grow isolated in LDC. Both CP-FL and LDC procedures proved efficient for generating high productive and stable cell clones. Overall productivity for individual clones depends on specific productivity, cell density and viability along time, allowing accumulation of the antibody. CP-FL clones reached maximum productivity at an earlier stage (2 weeks) of the 200 mL SF fed-batch experiment, which represents an advantage during the manufacturing process.

The 4 lead clones will be submitted to bioreactor runs to evaluate the most suitable clone for the Rebmab100 mAb to be used in clinical trials and eventually to go under production.
